# Cylindrical Fused Silica Resonators Driven by PZT Thin Film Electrodes with Q Factor Achieving 2.89 Million after Coating

**DOI:** 10.1038/s41598-019-45180-5

**Published:** 2019-07-01

**Authors:** Yiming Luo, Tianliang Qu, Yan Cui, Yao Pan, Menglin Yu, Hui Luo, Yonglei Jia, Zhongqi Tan, Jianping Liu, Bin Zhang

**Affiliations:** 1 College of Advanced Interdisciplinary Studies, National University of Defense Technology, 410073 Changsha, China; 2 Key Laboratory for Precision and Non-traditional Machining Technology of the Ministry of Education, Dalian University of Technology, 116024 Dalian, China

**Keywords:** Electrical and electronic engineering, Mechanical engineering

## Abstract

Cylindrical shell fused silica resonators coated with 8 axisymmetric Pb(Zr_0.53_Ti_0.47_)O_3_ (PZT) thin film electrodes (thickness ~2 *μm*) were reported. The resonators were firstly designed and fabricated, then annealed and processed by chemical etching to increase mechanical quality factor (Q factor) of resonators, which achieved as high as 2.89 million for n = 2 wineglass modes after being coated with PZT thin film electrodes. The n = 2 wineglass modes of the resonators were driven by PZT thin film electrodes in experiment and simulation with fine vibratory shape, which demonstrated the feasibility of the cylindrical fused silica resonator driven by PZT thin film electrodes. The application of PZT thin film electrodes to drive and detect cylindrical shell fused silica resonator can significantly improve Q factor of resonators and improve the sensitivity of Coriolis Vibratory Gyroscope (CVG).

## Introduction

Gyroscopes are key components of inertial navigation systems for detecting the angular motion of objects. Coriolis vibratory gyroscopes (CVGs) are simple in structure with no mechanical rotors, low energy loss, easy maintenance, high durability and high reliability. In the past few decades, the CVG has been studied extensively, including the hemispherical resonator gyroscope (HRG), the cylindrical resonator gyroscope (CRG), etc. Northrop Grumman’s HRGs have worked over 35 million hours without mission failure^1,2^. Meanwhile, the bias stability of the HRG developed by SAFRAN Inc. achieved better than 0.001 deg/h, and they have been widely used in different inertial systems successfully^3^. For high precision CVG, the high Q factor shell resonator is the key component. Both the hemispherical resonators produced by Northrop Grumman and SAFRAN Inc. have achieved the Q factor more than 10^7^ ^4,5^. By contrast, CRGs produced by Innalabs Inc. based on cylindrical shell resonators of metal materials only achieved a bias stability of 0.03–0.1 deg/h, owing to the Q factors of metal resonators, which are usually less than 10^5^ ^6,7^. In the field of micro CVG, micro birdbath resonator gyroscopes have improved their bias stabilities from 1 deg/h to 0.0391 deg/h with the Q factor nearly reaching 10^7^ ^8–10^. Meanwhile, self-induced parametric amplification has been introduced to increase the signal-to-noise ratio in micro resonating disk gyroscope^11^. High precision CVGs are usually equipped with high Q factor resonators because the drift characteristics of CVG pertain to the Q factor of resonators. However, compared with HRG, under the same processing precision (surface roughness, mass mismatch, concentricity tolerance, etc.) requirements, CRG has simpler mechanical processing, shorter manufacturing cycle and higher yield. Consequently, the development of cylindrical shell resonators with high Q factors can improve the precision of CRGs while reducing production costs significantly.

Resonators are usually made of metal, ceramic, fused silica or other materials. Among them, fused silica is ideal for shell resonators because of its excellent isotropic properties and extremely high Q factors. Previously we have presented monolithic cylindrical fused silica resonators with Q factors approaching 8 × 10^5^ ^12^. In comparison, the piezoelectric effect of PZT has been widely used in driving and detecting in CRGs because of its excellent frequency response characteristics. However, the Q factor of PZT materials is much lower than that of fused silica, and therefore the overall Q factor of cylindrical fused silica resonators will decrease drastically when PZT lamination electrodes (thickness more than 200 *μm*, including the top and bottom metal electrodes and the middle PZT lamination) are attached. We also experimentally verified that thinner PZT lamination electrodes were more conducive to maintain the Q factor at a higher value^13^. Moreover, PZT lamination electrodes are usually attached to the resonator by epoxy resin adhesives, which will also reduce the Q factor of resonators and degrade the performance of the CRG^14^.

The electrostatic driving scheme and piezoelectric driving scheme, both of which were designed to drive the resonators, have been illustrated in detail^15,16^. The electrostatic driving scheme has a sophisticated design with a high driving voltage (more than 100 V)^17^, resulting in an unsatisfactory driving efficiency. While the piezoelectric driving scheme has simplified mechanical structures and circuits, the Q factor of the resonators dropped dramatically after attaching PZT lamination electrodes^13^. Therefore, cylindrical shell resonators driven by PZT thin film electrodes were proposed and investigated through simulations and experiments in this paper, and the hypothesis that fused silica resonators can be driven effectively while maintaining a high Q factor after coating with PZT thin film electrodes was tested. PZT thin films have been researched extensively and widely used in the field of MEMS over the past few decades. In 1963, Foster of Bell LABS reported successfully producing an ultrasonic transducer by using CdS thin films^18^. In 1965, Foster and Rozgonyi created ZnO film transducers by reactively sputtering zinc in oxygen^19^. In 1979, Shiosaki *et al*. successfully attached AlN films to the substrate of glass and metal by rf reactive planar magnetron sputtering^20^. In 2004, Zinck *et al*. pointed out that the positive and inverse piezoelectric effect of PZT thin film electrodes can be used as vibration actuators in MEMS. They coated 800 nm of PZT thin film electrodes on a 10 *μm* silicon substrate, and using film actuation, the substrate produced a vibration displacement of 2 *μm*^21^. In 2010, Aktakka *et al*. reported the high performance of the piezoelectric energy collector based on PZT thin film electrodes. They also suggested the possibility of using PZT thin film technology to produce high-efficiency vibration actuators. In addition, they mentioned that reducing the dielectric loss and the mechanical damping of films improved the overall Q factor of the system^22^. However, to our knowledge, no articles have investigated the feasibility of coating PZT thin film electrodes on macro cylindrical shell resonators for the purpose of driving and detection.

In this paper, two types of resonators named Ge01 and Ge02 were designed and fabricated. Post-processing techniques such as annealing and chemical etching were utilized to improve the Q factor of resonators. The feasibility of cylindrical shell resonators driven by PZT thin film electrodes was investigated through simulations and experiments, aimed at reducing the Q factor drop rate of the resonator-films structure. We present, for the first time, a fused silica shell resonator with a Q factor of 2.89 million after coating with PZT thin film electrodes. Moreover, compared with the traditional technical scheme, the positioning accuracy of coating PZT thin film electrodes is higher, which may greatly reduce the asymmetry drift error and has potential to help develop the CRG with high precision, small size and low cost.

## Materials

### Structure design of resonators

**Figure 1:**
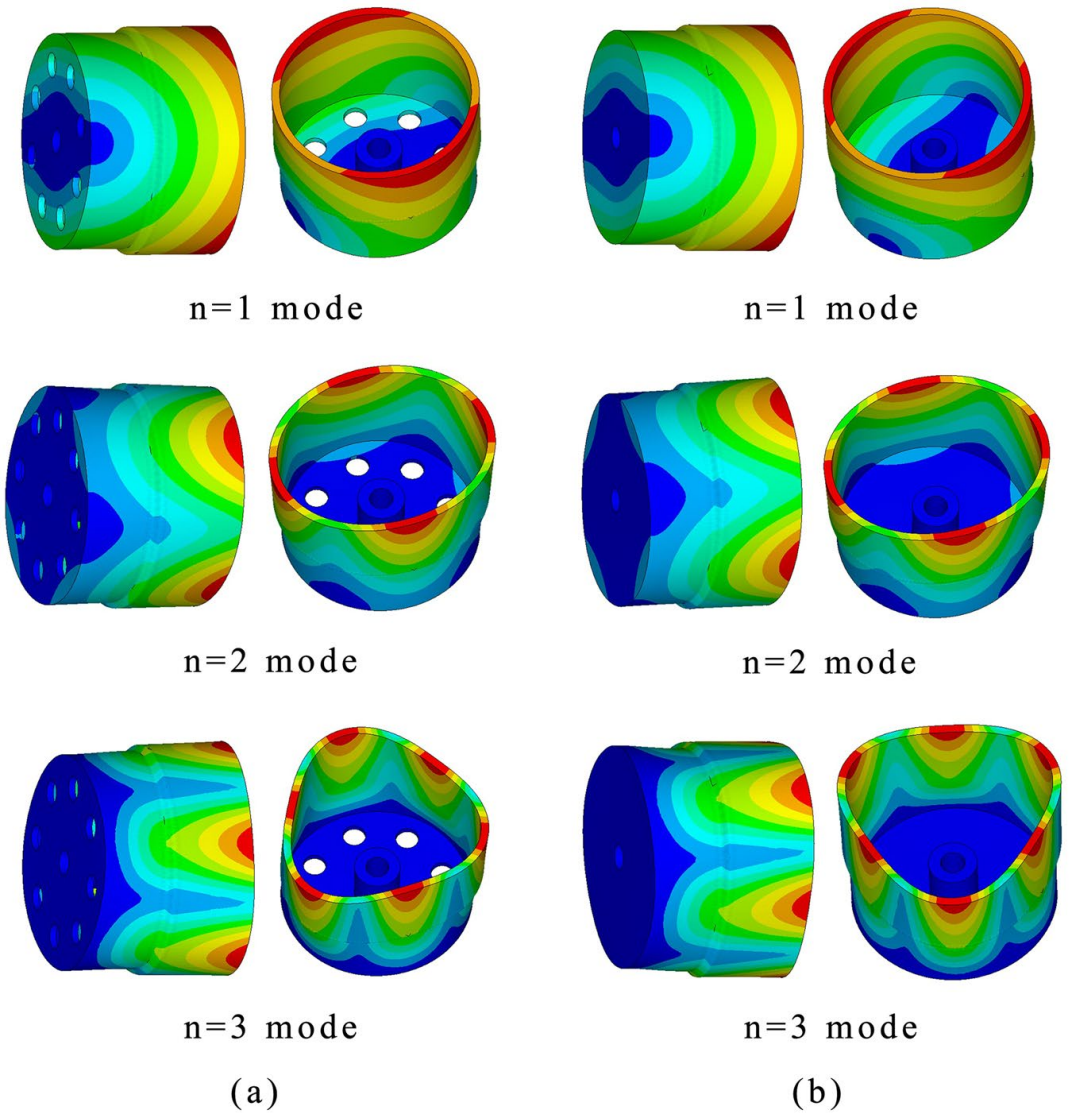
The model shapes of n = 1, 2 and 3 modes of resonators: (**a**) Ge01; (**b**) Ge02.

The structure of cylindrical fused silica resonators has been reported in ref.^12^. In order to adapt to the coating process of PZT thin film electrodes, we slightly changed the bottom structure of resonators based on the initial structure. For Ge01, we reduced the diameter of the bottom holes to 3 mm. For Ge02, we removed the bottom holes. Before the resonators were fabricated, the Ansys Multiphysics 14.0 software was employed to investigate the resonant frequency of resonators in n = 2 wineglass mode and its adjacent modes. The simulation results of n = 1 mode to n = 3 mode of Ge01 and Ge02 are depicted in Fig. 1. The resonant frequencies of n = 1 mode to n = 3 mode are listed in Table 1.

**Table 1:** The simulation results of resonant frequencies of resonators.

Resonator	Resonant frequency		
	n = 1 mode	n = 2 mode	n = 3 mode
Ge01	1363.9	4905.4	12684.1
Ge02	1419.3	4915.1	12692.1

The simulation showed that the frequencies of n = 2 wineglass mode were 4905.4 Hz (Ge01) and 4915.1 Hz (Ge02).

**Figure 2:**
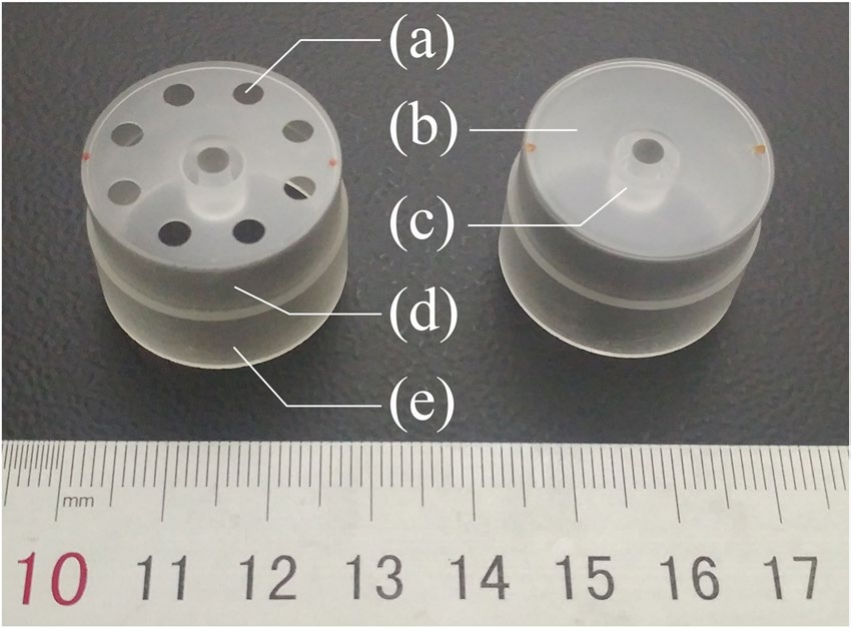
The structure of fused silica cylindrical resonators: (**a**) Bottom hole; (**b**) Bottom plate; (**c**) Anchor stem; (**d**) Vibration conducting shell; (**e**) Resonant shell.

The fabricated fused silica resonators are shown in Fig. 2, and the only difference between them is the presence of bottom holes. The sizes of the rest of the resonator are consistent with the original design: The diameter of resonators, height of the vibration conducting shell and height of the resonant shell were 25 mm, 8 mm and 10 mm, respectively.

### PZT thin films electrodes

**Figure 3:**
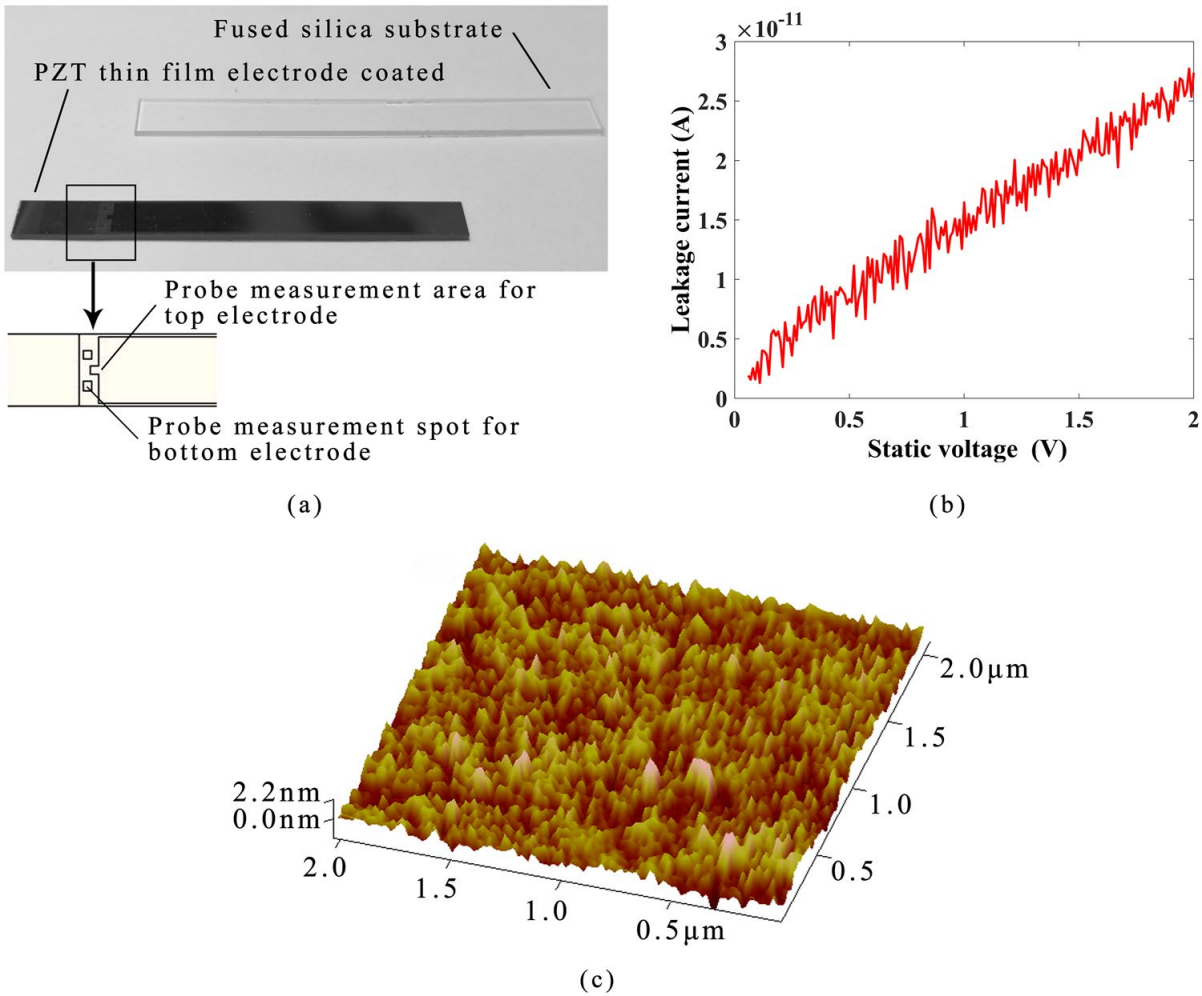
(**a**) The fused silica substrate before and after coated with the PZT thin film electrode; (**b**) Leakage current testing result of the PZT thin film electrode coated on the fused silica substrate; (**c**) The AFM scanned graph of the PZT thin film electrode in an area of 2 *μm* × 2 *μm*.

The film quality and the driving ability of PZT thin film electrodes were firstly investigated by coating the films onto a fused silica rectangular substrate. The length, width and thickness of PZT thin film electrodes (composed of the middle PZT layer, the top and bottom Ti/Pt electrode layers) were 19.6 mm, 4 mm and 2 *μm*, respectively, while those of the substrate was 25 mm, 4.5 mm and 1 mm, respectively. The fused silica substrates before and after coating are shown in Fig. 3(a). Ideally, the PZT layer acts as an insulator and changes shape when an electric field is applied. However, in the actual films, due to defects, there will be a tiny current in the PZT layer when an electric field is applied, which is the leakage current. On this account, the leakage current of the whole PZT thin film electrodes on the rectangular fused silica substrate was tested by a probe-type current meter. The results, as depicted in Fig. 3(b), showed that the leakage current increased with the applied static voltage, and the leakage current was on the level of 10^−11^ A under 2 V static voltage. In addition, for the purpose of further observing the surface quality of the PZT thin film electrodes, an atomic force microscope (AFM) was utilized to test its surface morphology. The surface morphology of an area of 2 *μm* × 2 *μm* is depicted in Fig. 3(c). According to the measured data, the Nanoscope Analysis software calculation results illustrated that the surface roughness of the PZT thin film electrodes was 7.76 nm.

## Experimental Results and Discussion

### Post-processing of resonators

For cylindrical fused silica resonators, the Q factor represents the ratio of energy lost in a motion cycle of vibration to the total energy of the resonators:

1 $${Q}=\frac{2{\rm{\pi }}E}{\Delta E}=\frac{2{\rm{\pi }}E}{{\sum }_{i=1}^{n}\Delta {E}_{{i}}},$$

where *E* represents the energy stored in the resonators, *ΔE*_*i*_ represents the energy loss in each cycle caused by the No. *i*^th^ factor (thermoelastic loss, air viscous damping, anchor loss, internal friction loss, surface loss, etc.^23–25^). Consequently, a higher Q factor indicates a lower overall loss of resonators. After initial processing of the fused silica resonators, a series of post-processing techniques, including annealing and chemical etching was utilized to increase the Q factor by reducing damping and loss. Besides, the bottom plate of the resonator needs mechanical polishing to meet the coating requirements.

**Figure 4:**
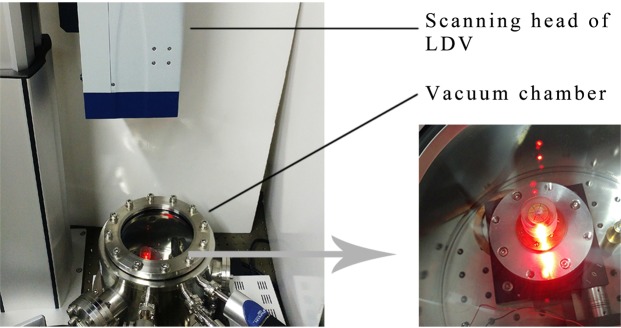
The vibration characteristics measuring system.

For the purpose of directly detecting the vibration characteristics (resonant frequency, resonant amplitude, vibrational shape, etc.) of resonators, measurements were made using a laser Doppler vibrometer (LDV) and a vacuum chamber, as shown in Fig. 4. Before and after coating with PZT thin film electrodes, the resonators were driven by acoustic waves and PZT thin film electrodes, respectively. The measurement steps have been illustrated in our previous study^12^. The decay time method was employed to calculate the Q factor. The decay time represents the time duration for the resonant amplitudes, *A*, decreasing to *A*/*e* (*e* ≈ 2.718):

2 $$Q=\pi f\tau ,$$

where *f* represents the resonant frequency in Hz and *τ* represents the decay time in seconds.

### Annealing

Annealing can reduce the internal stress of fused silica resonators due to mechanical grinding, thus greatly improving the Q factor^26–28^. It can also desorb oxygen molecules and water vapor in the air that adhere to the surface of resonators, and improves the Q factor by destroying the silica silanol (SiOH) bond which increases surface loss^29^. The resonant frequencies and Q factors of Ge01 and Ge02 were tested before and after annealing (driven by acoustic waves), under 1 atm. and 90 Pa in a vacuum chamber, as listed in Table 2.

**Table 2:** The testing results of the n = 2 mode of resonators before and after annealing.

Resonator	Before (as-fabricated)		After		
	Resonant frequency (Hz, 1 atm.)	Q factor (1 atm.)	Resonant Frequency (Hz, 1 atm.)	Q factor (1 atm.)	Q factor (90 Pa)
Ge01	5388.4	3093	5395.9	4053	199930
Ge02	5003.4	3822	5011.8	4466	82039

**Figure 5:**
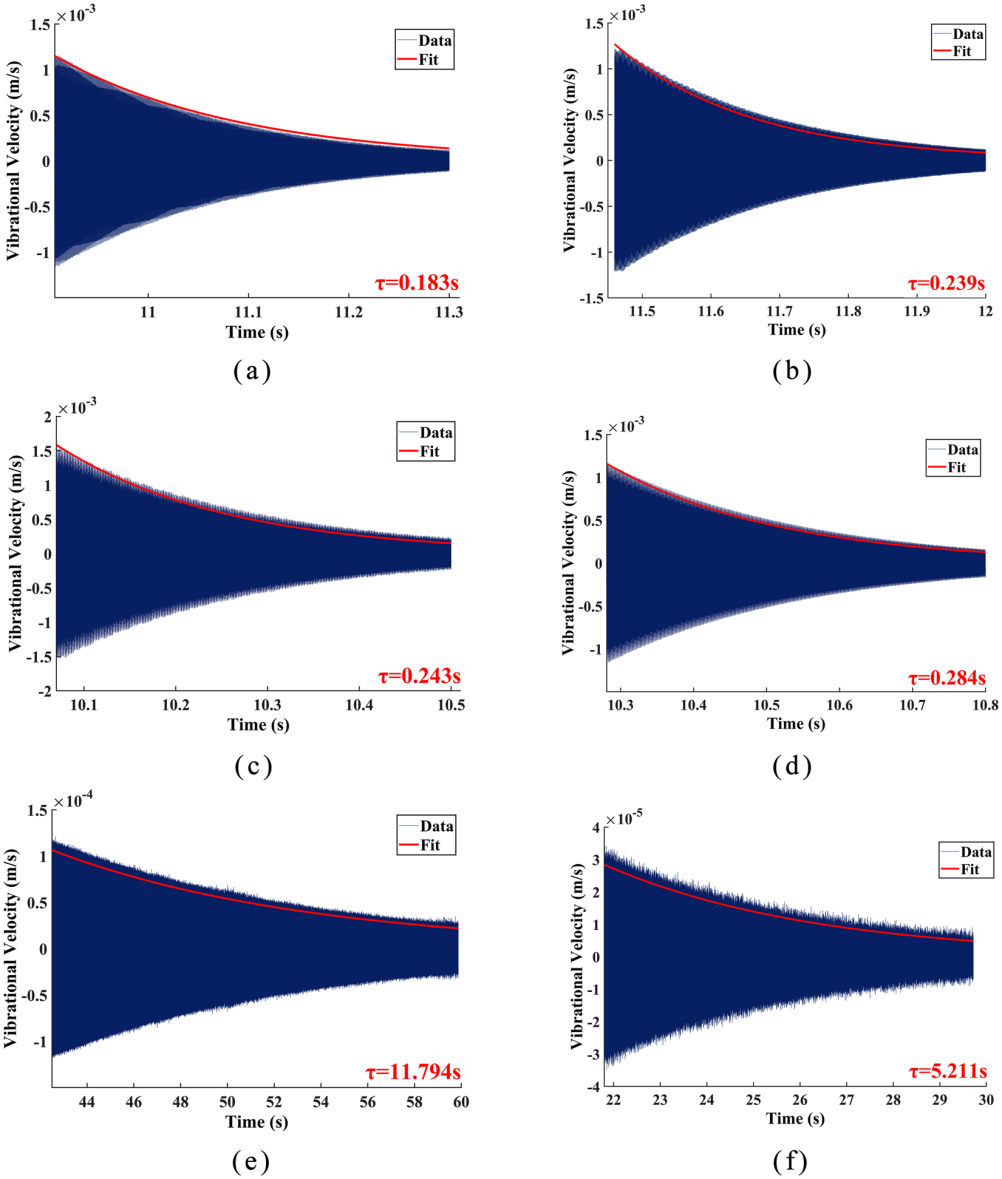
Decay time measurement of the two resonators: (**a**) Ge01 before annealing under 1 atm.; (**b**) Ge01 after annealing under 1 atm.; (**c**) Ge02 before annealing under 1 atm.; (**d**) Ge02 after annealing under 1 atm.; (**e**)Ge01 after annealing under 90 Pa; (**f**) Ge02 after annealing under 90 Pa.

During previous experiments, we found that when the atmospheric pressure was below 100 Pa, the resonators without any post-processing were unable to be driven by acoustic waves due to high losses and the insufficiency of acoustic waves’ driving force under low atmospheric pressure. Therefore, the Q factor of resonators before annealing was measured only under 1 atm. The vibration data acquired from the LDV were processed by MATLAB, and results are depicted in Fig. 5. The results showed that after annealing, the Q factor of Ge01 and Ge02 increased 31% (from 3093 to 4053) and 17% (from 3822 to 4466) under 1 atm., respectively. After the two resonators were put into a vacuum chamber to reduce the air viscous damping loss, the Q factors increased to 199930 (decay time 11.768 s) and 82039 (decay time 5.198 s) under 90 Pa, respectively. Moreover, annealing also slightly changed the resonant frequencies of Ge01 and Ge02, which increased by about 7.5 Hz and 8.4 Hz, respectively. It is worth noting that the measured values of resonant frequencies were different from that of simulation, as there are slight difference between the simulation input parameters and the actual material parameters, and errors in the machining process.

### Chemical etching

Chemical etching can remove the surface defect layer caused by machining of resonators by immersing resonators in suitable chemical solutions, so as to reduce the surface loss and improve Q factors^30,31^. The Q factors, resonant frequencies, mass variation and thickness variation of resonant shells after chemical etching are listed in Table 3.

**Table 3:** The testing results of the n = 2 mode of resonators after chemical etching.

Resonator	Resonant frequency (Hz)	10 Pa	Q factor (10 Pa)	Mass (g)	Removed mass (g)	Thickness decrease (mm, Measured at the resonant shell)
	1 atm.					
Ge01	5137.5	5149.1	1020922	2.5511	0.2778	0.047
Ge02	4677.8	4689.9	1572983	1.9065	0.2842	0.064

After chemical etching, the resonant frequencies of Ge01 and Ge02 decreased to 5137.5 Hz and 4677.8 Hz, respectively. The masses of Ge01 and Ge02 decreased to 2.5511 g (decreased by 0.2778 g) and 1.9065 g (decreased by 0.2842 g), and the thicknesses of the resonant shells decreased by 0.047 mm and 0.064 mm, respectively. The resonant frequencies were affected by both the structure’s stiffness and mass. However, while mass decreased linearly during chemical etching, the decrease in thickness led to a more significant drop of the structure stiffness and contributes more to the decrease in resonant frequencies^32^. Therefore, the combined effects of decreased mass and stiffness during chemical etching resulted in a decrease of resonant frequencies after chemical etching.

**Figure 6:**
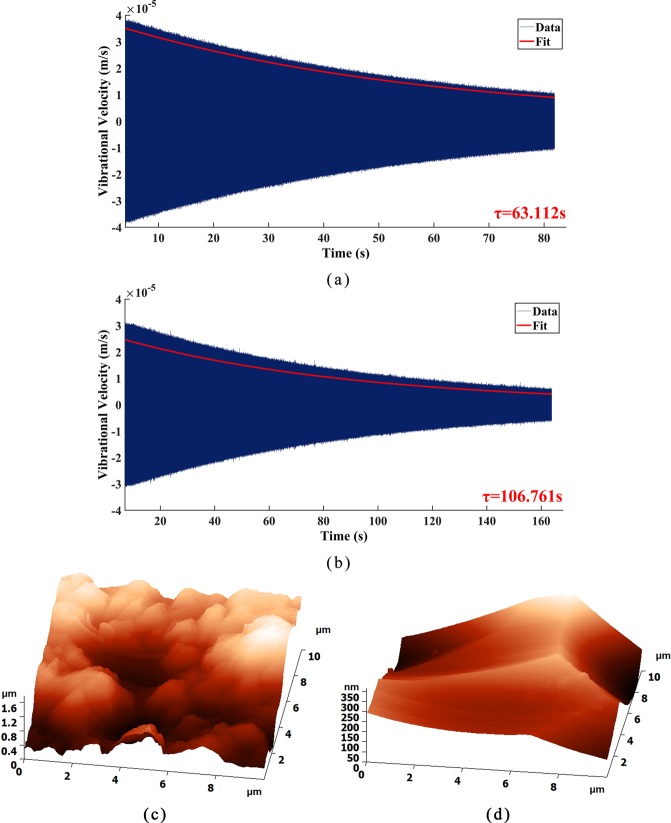
Decay time measurement of the resonators: (**a**) Resonator Ge01; (**b**) Resonator Ge02; Surface topography of resonator Ge02: (**c**) before chemical etching; (**d**) after chemical etching.

The pressure in the vacuum chamber decreased from 90 Pa to 10 Pa after chemical etching to further reduce the air viscous damping loss. When tested under 10 Pa (while other conditions remain unchanged), the resonant frequencies of Ge01 and Ge02 increased to 5149.1 Hz (increased by 11.6 Hz) and 4689.9 Hz (increased by 12.1 Hz), respectively. In addition, the Q factor of Ge01 increased to 1020922 with the decay time reaching 63.112 s, as depicted in Fig. 6(a), and the Q factor of Ge02 increased to 1572983 with the decay time reaching 106.761 s, as depicted in Fig. 6(b). Due to the corrosion of the etching solution on the surface of the resonator, the surface microcracks generated by the mechanical grinding process and the surface adsorption layer were removed. Consequently, the Q factor improved greatly after chemical etching. In order to further verify the surface promotion of resonators after chemical etching, the same surface of Ge02 before and after chemical etching was scanned at the area of 10 *μm* × 10 *μm* by AFM, as depicted in Fig. 6(c,d).

The AFM results demonstrated that, after chemical etching, the surface of the resonator was smoother than before. The surface roughness decreased from 1.611 *μm* to 0.367 *μm*, showing that chemical etching was an effective method to reduce the surface roughness of resonators.

After chemical etching, the surface roughness of bottom plate was still too large (over 100 nm) for film coating. A mechanical polishing process was introduced to further reduce the surface roughness of the bottom plates of the two resonators. The resonant frequencies of Ge01 and Ge02 were decreased to 5090.3 Hz and 4658.4 Hz, respectively, after mechanical polishing.

### Simulation of PZT thin films electrodes

The PZT thin film electrodes were mainly used in micro-mechanical structures. However, the cylindrical shell resonator was a macro-mechanical structure. The thickness of the PZT thin film electrodes was less than 2 *μm*, less than 1% of that of the PZT lamination electrodes. Hence the driving force provided by the PZT thin film electrodes is limited and smaller than that of the PZT lamination electrodes, with the result that the driving energy provided by the PZT thin film electrodes has to be larger than the loss in order to actuate and to keep the resonator in a stable resonant amplitude. Consequently, a high Q factor was needed, indicating the low loss of resonators. After post-processing, the Q factors of both Ge01 and Ge02 were over 1 million, making it possible to drive resonators using PZT thin film electrodes. In addition, it is necessary to simulate the vibration amplitude of resonators driven by PZT thin film electrodes to verify its feasibility. The vibration amplitude (denoted by *A*) of the resonator is directly proportional to the driving force (denoted by *F*) in the unit area and the Q factor of the resonator^33^:

3 $$A\propto F\cdot Q$$

**Figure 7:**
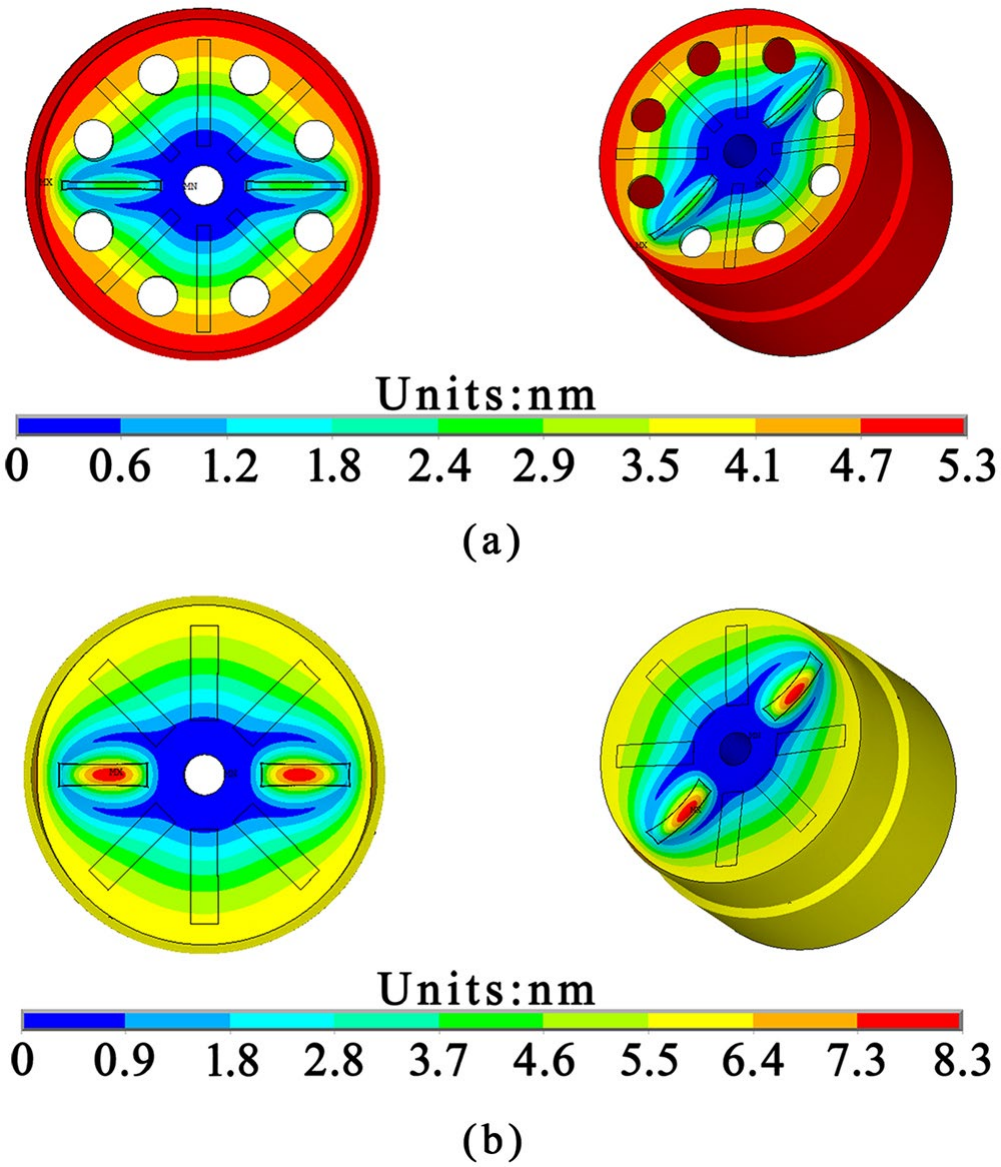
Amplitudes simulation results of the Ge01 and Ge02 driven by PZT thin film electrodes.

The Ansys Multiphysics 14.0 software was employed to investigate the static deformation of resonators, and the results are depicted in Fig. 7.

The simulation results verified the feasibility of driving resonators with PZT thin film electrodes, and the maximum displacement at the bottom edge of the resonator Ge01 and Ge02 were 5.3 nm and 7.3 nm, respectively. In addition, the amplitude is larger when driving the resonators under sinusoidal voltage at the resonant frequency.

### Coating experiments of PZT thin film electrodes on resonators

**Figure 8:**
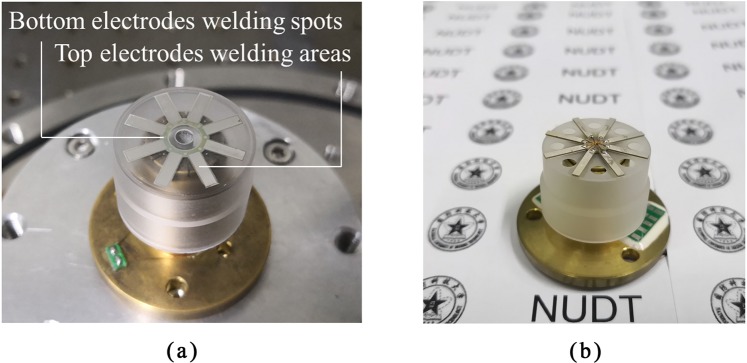
(**a**) Ge02 coated with PZT thin films electrodes; (**b**) MacroPR attached with PZT lamination electrodes.

The sol-gel method was utilized to coat PZT thin film electrodes on Ge01 and Ge02. During coating, the films on Ge01 cracked due to the edge-bead effect between the eight bottom holes. The holes also caused complex liquid flow directions and a non-uniform film thickness. Consequently, the films failed to work properly under the driving voltage. In contrast, eight PZT thin film electrodes were coated on the bottom plate of Ge02 successfully, as shown in Fig. 8(a). The coating experiment results demonstrated that resonators without bottom holes were more suitable for coating high-quality PZT thin film electrodes. The thickness, length and width of the PZT thin film electrodes were 2 *μm*, 7 mm and 2 mm, respectively. Eight reserved bottom electrode welding spots were grounded, and eight top electrodes were used to load the driving signals or output the detection signals after welding with wires.

In our previous experiment, the PZT lamination electrodes (thickness ~200 *μm*) were attached on the fused silica resonator (named as MacroPR), and the result illustrated that the Q factor of the resonator decreased by 96.9% (tested under 50 Pa), which was detrimental to maintaining the high Q factor of resonators. The PZT lamination electrodes attached to the fused silica resonator are shown in Fig. 8(b).

Both Ge02 and MacroPR were driven by acoustic waves during the test. The testing results of Ge02 and MacroPR driven by acoustic waves were listed in Table 4.

**Table 4:** The testing results of the n = 2 mode of MacroPR and Ge02 after PZT lamination electrodes attached and PZT thin film electrodes coated, respectively.

Resonator	Resonant frequency (Hz)		Q factor		Drop rate
	before	after	before	after	
MacroPR	3157.7	3163.4	487279	15019	96.9%
Ge02	4658.1	4658.4	1572983	1026877	34.7%

After coating, the resonant frequency of Ge02 changed slightly (from 4658.4 Hz to 4658.1 Hz), because the mass of the PZT thin film electrodes was very small compared with that of the resonator. Additionally, the Q factor of Ge02 decreased to 1026877 (drop rate: 34.72%, tested under 10 Pa). The drop rate is much lower than that of MacroPR.

### Testing of PZT thin film electrodes driving capability

**Figure 9:**
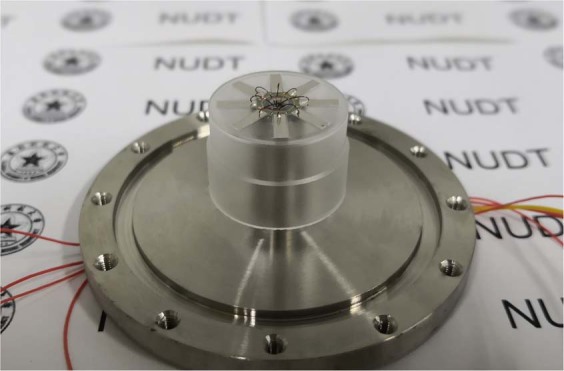
Ge02 fixed on the Invar base and conductor wires completed.

**Figure 10:**
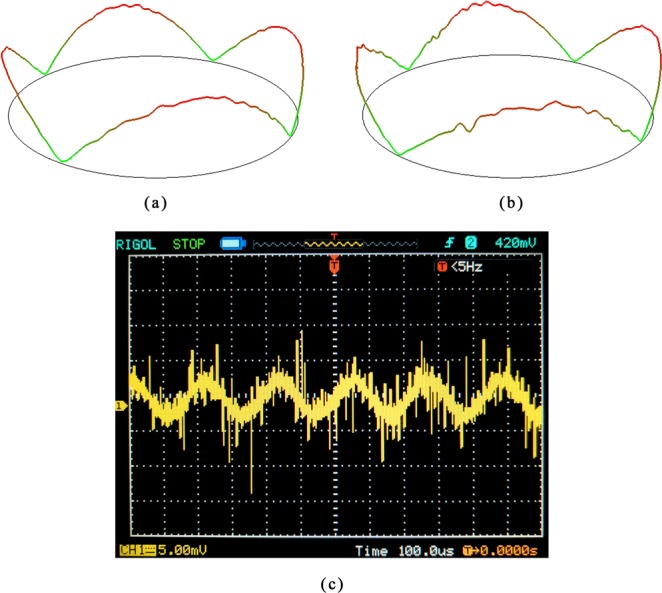
The vibration shape comparison: (**a**) Driven by acoustic waves; (**b**) Driven by PZT thin film electrodes; (**c**) Output voltage signal of PZT thin film electrodes on detection axis.

The simulation results depicted in Fig. 7 have verified the driving feasibility of PZT thin film electrodes. Epoxy was used to glue the resonator on an Invar base to reduce anchor loss, and conducting resin was used to glue wires to the top and bottom electrodes of the films before testing, as shown in Fig. 9. We tested the resonant amplitudes of Ge02 driven by PZT thin film electrodes under 2.6 × 10^−2^ Pa. The results are listed in Table 5. The resonant shape of Ge02 driven by acoustic waves and PZT thin film electrodes are depicted in Fig. 10(a) and (b), respectively, while the output signal of PZT thin film electrodes detected with an oscilloscope is depicted in Fig. 10(c). It is worth pointing out that, for a mature CRG product, PZT thin film electrodes are used both for driving the resonators and detecting signals with a direct read-out system. But so far in our initial experimental research, detecting by LDV has the advantage of easily detecting the vibration shape and amplitude, which are the key parameters during the developmental stage.

**Table 5:** The resonant amplitudes testing results of the n = 2 mode of Ge02 under 2.6 × 10^−2^ Pa with different driving sinusoidal voltage at the resonant frequency.

Resonator	Driving voltage (V)	Resonant amplitude (nm)
Ge02	1	7.75
	2	9.84
	3	14.77

The shape graphs in Fig. 10 were obtained by scanning the bottom edge of the resonator through LDV. The results demonstrated that the macro fused silica cylindrical resonator can be driven by PZT thin film electrodes successfully. When Ge02 was driven by PZT thin film electrodes under the sinusoidal voltage at 3 V (peak to peak value), the resonant amplitudes reached 14.77 nm, and the output detection signal of the PZT thin film electrodes reached about 10 mV (peak to peak value). The results showed that the driving and detection by PZT thin film electrodes can maintain the resonator in a stable working mode. To prevent the films from breaking down, the driving voltage cannot be overly high. In the future work, we will improve the coating process to improve the breakdown voltage threshold.

**Figure 11:**
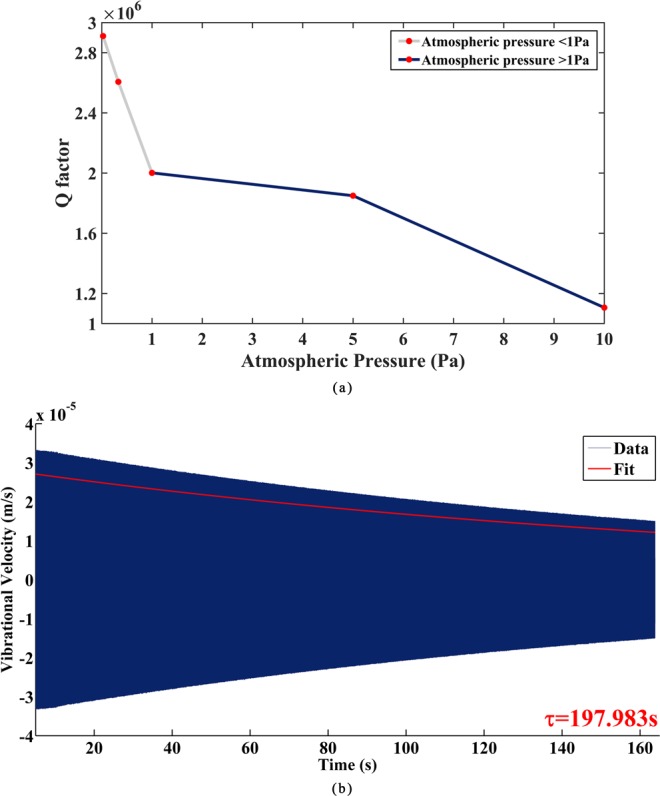
(**a**) The varies of Q factor with different atmospheric pressures; (**b**) Decay time measurement of Ge02 after coated with PZT thin film electrodes.

To analyze the influence of air viscous damping on Q factor, we tested the resonant amplitudes of Ge02 driven by PZT thin film electrodes under different pressure with the sinusoidal voltage at 3 V. The results are listed in Table 6, and the variation of Q factors with different atmospheric pressures are depicted in Fig. 11(a). The decay time under 2.6 × 10^−2^ Pa is depicted in Fig. 11(b).

**Table 6:** The Q factor testing results of the n = 2 mode of Ge02 under different pressures.

Resonator	Pressure (Pa)	Resonant frequency (Hz)	Q factor
Ge02	10	4652.18	1106048
	5	4652.11	1849767
	1	4652.07	2001502
	3.3 × 10^−1^	4652.05	2606583
	2.6 × 10^−2^	4651.98	2893447

The results illustrated that when the atmospheric pressure was less than 1 Pa, the growth rate of the Q factor as pressure decreased was much higher than that when pressure was higher than 1 Pa, which indicated that further decrease of the atmospheric pressure can lead to more significant improvement of the Q factor. Under 2.6 × 10^−2^ Pa, the Q factor of Ge02 reached 2893447 with the decay time of 197.983 s. It is worth noting that due to the limitation of the maximum measurement range of LDV, the decay time was obtained by sinusoidal exponential fitting the measurement data. To the best of our knowledge, this is by far the highest Q factor of macro cylindrical resonators with actuators and detectors attached.

## Conclusions

Fused silica cylindrical shell resonators driven by PZT thin film electrodes was investigated, and two novel structures of resonators were designed and fabricated to meet the requirements of film coating. The results demonstrated that the resonator without bottom holes was more suitable for coating with PZT thin film electrodes. Through annealing and chemical etching, we successfully increased the Q factors of both two resonators to more than 1 million. Moreover, the feasibility of macro cylindrical shell fused silica resonators driven by PZT thin film electrodes was verified through simulations and experiments. The resonator driven by PZT thin film electrodes under sinusoidal voltage at 3 V achieved a vibration amplitude of 14.77 nm with the output detection signal reaching about 10 mV (peak to peak value). Finally, we demonstrated that when the atmospheric pressure was lower than 1 Pa, the Q factor increased remarkably as pressure decreased, and the Q factor of the cylindrical resonator reached 2.89 million after coating with PZT thin film electrodes under 2.6 × 10^−2^ Pa. This is the highest Q factor of the macro cylindrical shell resonator reported to date. It is worth noting that the Q factor was not measured under the same atmospheric pressure, because the Q factor of the resonator was not high enough before post-processing, with the result that it could not be driven by acoustic waves under a lower atmospheric pressure. To measure the improvement of the Q factor in each step, the final atmospheric pressure (2.6 × 10^−2^ Pa) could not be set at the beginning. For a mature CRG product, the internal atmospheric pressure was around 10^−5^ Pa. Consequently, a higher Q factor can be obtained assuming that loss due to air viscous damping can be further decreased. Further research will be focused on the investigation and development of the PZT thin film electrodes read-out system, including output signal preamplifier circuit, temperature compensation and frequency feedback circuit. Once completed, a new solution for driving and detecting of CRG may be provided based on PZT thin film electrodes with lower driving voltage, simpler mechanical structure and control circuits. The inherent monolithic structure proposed in this paper can effectively improve the accuracy of CRG and reduce the system power consumption, which potentially can be used in sea-based, land-based and space-based equipment.

## Methods

### Simulation of resonators

Ansys Multiphysics 14.0 software was utilized to perform simulation on fused silica resonators. During the simulation of resonators driven by PZT thin film electrodes, eight 2 *μm* PZT thin film electrodes were attached to the bottom plates of the resonators. Two axisymmetric PZT thin film electrodes were applied with a 3 V static voltage, which was used for driving the resonators. The length and width of PZT thin film electrodes designed for Ge01 were 8 mm and 1 mm, respectively, and 7 mm and 2 mm for Ge02, respectively.

### Manufacturing and post-processing of resonators

The manufacturing steps of resonators have been detailed in ref.^12^. The annealing procedure has been optimized compared with our previous work^12^. An annealing furnace equipped with a vacuum tube was utilized to perform annealing experiments on resonators, which was first heated to 800 °C with a rate of 10 °C/min and then 4 °C/min to 1150 °C. During the heat preservation process, both Ge01 and Ge02 were annealed under a temperature of 1150 °C for 12 hours. After that, the resonators were cooled at a rate of 0.5 °C/min to 900 °C, followed by 1 °C/min to 800 °C, then allowed to cool to room temperature naturally. The atmospheric pressure was 10^−3^ Pa during annealing.

Before the chemical etching process, Ge01 and Ge02 were fixed with a joystick through the central hole on the bottom plate, and then immersed in an etching solution of 10:1 in volume ratio of NH_4_F (40 wt %) and HF (40 wt %) for 30 minutes. The temperature of the solution was maintained at 60 °C during the etching process. To prevent the solution concentration around the resonators from changing dramatically due to the reaction, a magnetic stirrer was added into the solution container, which caused the solution to circulate uniformly. The mass of the resonators and the thickness of the resonant shells were measured by an electronic balance and a measuring microscope produced by Werth Inc. after chemical etching.

### PZT thin film electrodes fabrication

**Figure 12:**
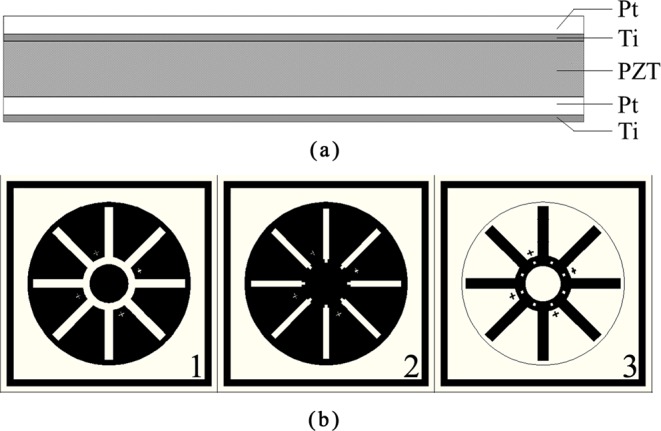
(**a**) Designed structure of PZT thin film electrodes; (**b**) Designed structure of mask plates.

The designed structure of the driving and detecting thin film electrodes are shown in Fig. 12(a) with a thickness of 2 *μm*. The PZT thin film electrodes were composed of five film layers, which are Ti (~50 nm), Pt (~200 nm), PZT (~1.5 *μm*), Ti (~50 nm), Pt (~200 nm) from bottom to top, respectively.

The PZT layers and the Ti/Pt layers were coated using the Sol-Gel method and the magnetron sputtering method, respectively. The PZT layers were made from lead zirconate titanate (Pb[Zr_0.53_Ti_0.47_]O_3_) which is composed of lead acetate (Pb(CH_3_COOH)_2_•H_2_O), zirconium nitrate (Zr(NO_3_)_4_•5H_2_O), tetrabutyl titanate (Ti(OC_4_H_9_)_4_), ethylene glycol methyl ether (CH_30_C_2_H_4_OH) and acetylacetone (CH_3_COCH_2_COCH_3_). The fabrication process of the PZT thin film electrodes has been illustrated in our previous study^34^. In order to obtain the desired film shape, three mask plates, as shown in Fig. 12(b), were used for the bottom Ti/Pt layers (first plate), the top Ti/Pt layers (second plate) and the PZT layer (third plate), respectively.

## Data Availability

All raw data analyzed or measured in this paper are available from the corresponding author, upon receipt of a reasonable request.
